# A monoclonal antibody marker for the exclusion-zone filaments of *Trypanosoma brucei*

**DOI:** 10.1186/1756-3305-1-21

**Published:** 2008-07-10

**Authors:** Mélanie Bonhivers, Nicolas Landrein, Marion Decossas, Derrick R Robinson

**Affiliations:** 1 UMR-CNRS 5234, University of Bordeaux, 233076 Bordeaux, France; 2 CNRS-UPR9021-IBMC, 67084 Strasbourg, France

## Abstract

**Background:**

*Trypanosoma brucei *is a haemoflagellate pathogen of man, wild animals and domesticated livestock in central and southern Africa. In all life cycle stages this parasite has a single mitochondrion that contains a uniquely organised genome that is condensed into a flat disk-like structure called the kinetoplast. The kinetoplast is essential for insect form procyclic cells and therefore is a potential drug target. The kinetoplast is unique in nature because it consists of novel structural proteins and thousands of circular, interlocking, DNA molecules (kDNA). Secondly, kDNA replication is critically timed to coincide with nuclear S phase and new flagellum biogenesis. Thirdly, the kinetoplast is physically attached to the flagellum basal bodies *via *a structure called the tripartite attachment complex (TAC). The TAC consists of unilateral filaments (within the mitochondrion matrix), differentiated mitochondrial membranes and exclusion-zone filaments that extend from the distal end of the basal bodies. To date only one protein, p166, has been identified to be a component of the TAC.

**Results:**

In the work presented here we provide data based on a novel EM technique developed to label and characterise cytoskeleton structures in permeabilised cells without extraction of mitochondrion membranes. We use this protocol to provide data on a new monoclonal antibody reagent (Mab 22) and illustrate the precise localisation of basal body-mitochondrial linker proteins. Mab 22 binds to these linker proteins (exclusion-zone filaments) and provides a new tool for the characterisation of cytoskeleton mediated kinetoplast segregation.

**Conclusion:**

The antigen(s) recognised by Mab 22 are cytoskeletal, insensitive to extraction by high concentrations of non-ionic detergent, extend from the proximal region of basal bodies and bind to the outer mitochondrial membrane. This protein(s) is the first component of the TAC exclusion-zone fibres to be identified. Mab 22 will therefore be important in characterising TAC biogenesis.

## Background

*T. brucei *has a highly ordered polar cytoskeleton [1-3]. Cytoskeleton morphogenesis is central to cell cycle events such as organelle positioning, segregation, mitosis and cytokinesis [3-5]. Trypanosome parasites also have a precise order, location and organisation of microtubules and filaments that interact with essential single copy organelles including the flagellum, mitochondrion, mitochondrion genome (kinetoplast) and Golgi apparatus [2,4,6,7]. The flagellum has roles in controlling cell length, kinetoplast segregation, cell attachment to tsetse fly salivary glands and motility. Furthermore, recent data has shown that flagella motility is indispensable in bloodstream forms [4,5,8]. The only protein that provides data on the molecular nature of the cytoskeletal component(s) responsible for positioning of the kinetoplast is p166, an essential component of the TAC [9]. The p166 protein is located in the mitochondrion on the matrix side of the inner mitochondrial membrane and adjacent to the kinetoplast. Knockdown of p166 by RNA interference (RNAi) induces profound effects on kinetoplast segregation resulting in kinetoplast networks that replicate without segregating and thus forming huge networks that are up to ten times larger than unit sized networks. The TAC appears to be present throughout the cell cycle, which means that kinetoplast S phase occurs whilst the kinetoplast is attached to the basal bodies and is simultaneous with basal body separation. After kDNA replication, one of the daughter kinetoplast is repositioned by separation of the basal bodies of the new flagellum, which in turn, is a microtubule-based mechanism – thus posing the question what are the structural components of the TAC? Here we present a novel electron microscope technique for visualizing *T. brucei *cells after mild detergent extraction but allowing the retention of intact mitochondrial membranes. We also present the cellular and biochemical characterisation of the monoclonal antibody Mab 22, which is the first marker for the exclusion zone filaments of the TAC. Mab 22 will be essential in studies concerning TAC organization, function and biogenesis.

## Methods

### Immuno-electron microscopy

4 ml of mid-log-phase EATRO 1125 wild-type procyclic cells were collected by centrifugation, 5 min 1,000 × g. The supernatant was discarded and the cells were gently resuspended and washed in 4 ml phosphate buffered saline (PBS) pH 7.2. The cells were centrifuged as above and resuspended in 200 μl PBS then adhered onto a 10 well glass slide in 20 μl drops/well. The slide was previously coated with poly-L-Lysine for 10 min as used in standard immunofluorescence techniques – see [10]. Cells were permeabilised by the addition of 50 μl droplets/well of 0.025% Triton T× 100 in 100 mM PIPES, 2 mM EGTA, 2 mM MgSO4, 0.1 mM EDTA, pH 6.9 (PEME) for 3 min at room temperature. Cells were then washed with 50 μl droplets/well of PEME for 5 min and then fixed in 4% paraformaldehyde, 0.2% glutaraldehyde in PEME (25 μl droplets/well) for 4 min. Free aldehyde groups were blocked by the addition of 25 μl droplets/well of 100 mM glycine in PEME 2 × 10 min, followed by 25 μl droplets/well of PEME, 5 min. The cells were then blocked with 50 μl droplets/well in PBS, 2% BSA, 0.1% Tween 20 for 5 min, then incubated in 15 μl/well of Mab 22 undiluted, or with PBS alone, or a control antibody (anti-PFR2 – L8C4 a kind gift from Keith Gull University of Oxford) for 2 hr in a sealed, moist and dark environment (an aluminium foil covered Petri-dish, with a PBS soaked tissue placed next to the slide). Cells were then washed with 50 μl droplets/well PBS, 2% BSA, 0.1% Tween 20, 0.01% sodium azide, 5 min, followed by 50 μl droplets/well PBS for 5 min. They were then incubated in 20 μl droplets/well with Goat Anti-mouse-IgM antibody coupled to Ultra-small gold particles (0.8 nm; Aurion, The Netherlands), diluted 1:100 (for the Mab 22 antibody) or (1:50 for the L8C4 antibody) in PBS, 2% BSA-c (acetylated BSA, Aurion), 1% fish skin gelatin, 0.01% sodium azide (Aurion – The Netherlands).

After secondary labelling the cells were washed in 50 μl droplets/well with PBS, 2% BSA-c, 1% fish skin gelatin, 0.01% sodium azide, 5 min, followed by 50 μl droplets/well with PBS, 0.01% sodium azide 5 min. They were then fixed in 25 μl droplets/well of 2.5% glutaraldehyde in PBS, 10 min, washed in PBS, 5 min, then washed again in 25 μl droplets/well acetate buffer 100 mM, pH 7.0, 5 min. The gold particle signal was enhanced using a silver enhancement kit (25 μl droplets/well HQ silver; Nanoprobes, NY, USA) for 7–11 min at RT in the dark. The reaction was stopped by three 5 min washes in acetate buffer, 50 μl droplets/well. Samples were briefly washed (30 sec) with PB buffer (160 mM Na_2_HPO_4_.2H_2_O, 40 mM NaH_2_PO_4_.2H_2_O, pH 7.4), 50 μl droplets/well then post-fixed 10 min with 1% OsO_4 _in PB (40 μl droplets/well).

After osmication cells were twice washed for 5 min with PB buffer, 50 μl droplets/well and the whole slide immersed or flooded in 50% ethanol (10 min). The dehydration step was repeated and the slide was immersed or flooded with 1% uranyl acetate in 75% ethanol for 25 min in the dark. The slide was immersed or flooded in 95% ethanol (10 min) followed by 100% ethanol (twice 10 min). A drop of freshly made resin ~50 μl (DURCUPAN, SIGMA) was added to each well and a gelatine capsule (EMS, 8.18 mm diameter or from a local pharmacy) filled with resin was inverted on each well. The resin was polymerised at 65°C for 48 h. Blocks were removed from the slide by gently heating the opposite face of the slide over a Bunsen burner for 1–3 sec. Ultrathin sections were cut, collected on pioloform-coated single-slot copper grids, stained with 2.7% lead citrate, and examined with a Technai electron microscope (FEI, The Netherlands). Negatives were scanned using a Polaroid SprintScan 45 Ultra negative scanner. Images were displayed and processed using Adobe Photoshop 8.

### Immunofluorescence

Immunofluorescence on cytoskeletons was as in [10] with dilutions in PBS of 1:10 for Mab 22, 1:500 for YL1/2 followed by Oregon green 488, anti-mouse FITC and Alexa Fluor 594 anti-rat conjugated secondary antibodies at 1:100 dilution in PBS. Or double immunolabelling was done as described in [11] with dilutions for Mab 22 1:10 and YL1/2 1:500.

### Cloning and expression of proteins

Tb11.01.3960 (*BILBO1*), Tb927.8.6660 (*P69*) and Tb11.01.2310 (*P99*) ORFs were amplified by PCR and cloned into the TOPO Blunt vector (Invitrogen). After checking the constructs by sequencing, the genes were sub-cloned into the bacterial over-expression vector pET28a(+) (Invitrogen) to express an N-terminal 6 histidine-tag thus producing pHis3960, pHis2310 and pHis6660 respectively. Tb11.01.2310 ORF was also cloned into the *T. brucei *expression vector pLew79-GFP1 [12] to express *in vivo *a C-terminal-GFP-tagged protein to produce p2310GFP. Constructs for BILBO1-GFP and RNAi are published in [10].

### Western blotting

Western blots were done as described in Bonhivers et al (2008) [10] using the mouse monoclonal Mab 22 1:5 dilution in blocking solution or the mouse monoclonal anti-polyhistidine (Sigma H-1029) diluted 1:3000. A rabbit polyclonal affinity purified antibody, anti-p99, was raised against two peptides of protein p99 (H2N-GPLRRDLDAYDEHVRR-CONH2 and H2N-QAVAEHGYHYVLPRIS-CONH2) (Eurogentec). Anti-p99 was used on blots of proteins from a 10% SDS-PAGE gel using 2.10^7 ^p6His99 expressing bacteria per well induced for 3 h with 1 mM IPTG. Wild type *T. brucei *whole cells were used at 1.10^7^/well and p99 RNAi non-induced or induced whole cells were used at 4.10^7^/well. Anti-p99 was used in the following dilutions PI: Pre-immune serum 1:1,000. I: Immune serum 1:500, AP: affinity purified immune serum 1:1000 in blocking solution. After membrane stripping, the L8C4 anti-PFR2 monoclonal antibody was used at 1:1000 dilution in blocking solution.

## Results

### Mab 22 recognizes basal body structures

**Figure 1:**
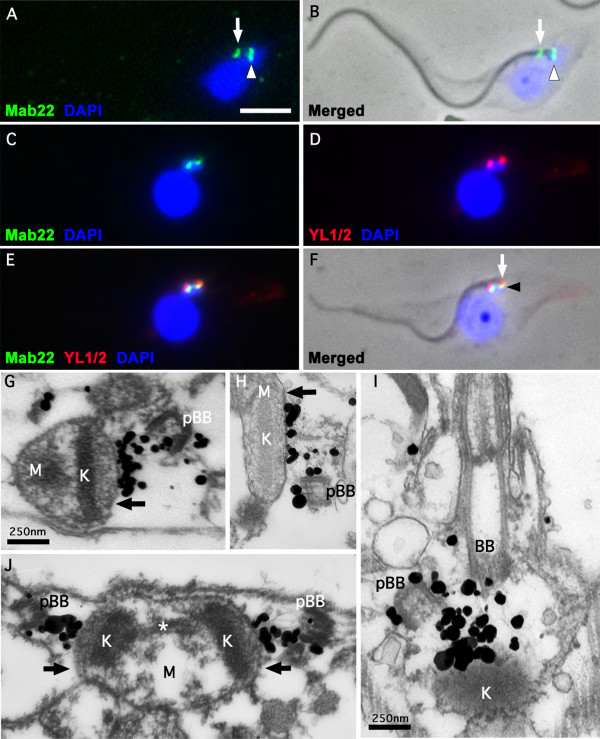
**Immunofluorescence and Immuno-electron microscope labelling of the *T. brucei *TAC**. A-F. Immunofluorescence on wild-type cytoskeletons. A. DAPI staining (blue) and Mab 22 labelling (green). B. A merged phase contrast, DAPI staining and Mab 22 labelling of the cytoskeleton in A. The arrows in A and B show the Mab 22 flagellar pocket collar label observed when this monoclonal was initially made and presumably had a very high titre. The arrowhead of A and B indicate the TAC exclusion zone label of Mab 22. C-D. A double labelling immunofluorescence micrograph illustrating a cytoskeleton showing Mab 22 and YL1/2 label. C. DAPI staining (blue) and Mab 22 labelling (green). D. DAPI staining (blue) and anti-basal body staining using YL1/2 (red). E. Merged of C and D. F. Phase contrast merged of E. The arrow in F illustrates the basal body labelling of YL1/2 and the arrowhead indicates the TAC exclusion zone label. In A-F scale bar is 5 μm. G-J. Electron micrographs of longitudinal sections of extracted and Mab 22 probed cytoskeletons. In all images the section traverse a basal and/or pro-basal bodies (BB, pBB). The immunolabelling is clearly observed between the basal or probasal bodies and the outer mitochondrion membrane (black arrow). Kinetoplast is marked as (K) and mitochondrion matrix marked as (M). In J, the kinetoplast is in early kinetoplast S phase and illustrates the presence of unilateral filaments and attachment to the mitochondrion membrane and kinetoplast. The asterisk in J denotes the "nabelschnur", a filamentous structure rich in basic proteins that links the kDNA discs during their segregation [18]. In G-J scale bar is 250 nm.

Our research has focused on identifying and functionally characterising minor *T. brucei *flagellar cytoskeleton proteins that have essential roles in cytoskeleton biogenesis. Upon immunising mice with flagella extracts of *T. brucei *EATRO wild-type 1125 procyclic cells [13], we made a number of monoclonal antibodies. One of these antibodies, Mab 22 (IgM), when used on methanol or paraformaldehyde fixed cytoskeletons gave an unusual, extreme distal, basal body immunofluorescence signal (See white arrowhead in Figure 1A and 1B). When cytoskeletons or flagella were probed simultaneously with Mab 22 and YL1/2 [14], a rat monoclonal antibody raised against tyrosinated tubulin, the Mab 22 immunofluorescence signal was proximal to the labelling patterns of YL1/2 (Figure 1C–F). Since YL1/2 identifies the aggregation of unpolymerized tyrosinated tubulin present in the transitional fibres of the mature flagellum [15], we realized that this result clearly pointed to the first identification of previously uncharacterised structures of the basal bodies, namely the TAC. Consequently, this result implicates the Mab 22 positive proteins as strong candidates for discrete components of the TAC. More precisely, these fibres are likely to be **exclusion-zone filaments **that traverse the cytoplasm from the basal bodies and link to the mitochondrion outer membrane thus physically binding the basal bodies to the mitochondrion. Immunofluorescence data also suggests that Mab 22 detects antigens found on both the mature and the pro-basal bodies. Indeed, two or more bright signals are observed on the basal bodies of G1 or post S phase cells respectively (See white arrowheads in Figure 1A and 1B). A weak flagellar pocket collar signal was also observed using this monoclonal when it was initially made (See white arrows in Figure 1A and 1B). The presence of this secondary signal was surprising since the hybridoma cell line producing the MAb 22 monoclonal had been cloned three times.

### Mab 22 binds to exclusion zone filaments of the TAC

To analyse further the unusual TAC signal observed with Mab 22 by immunofluorescence we developed a pre-embedding electron microscope labelling technique. This entails a limited detergent extraction of cells that allow Mab 22, followed by ultra-small gold particles, to infiltrate cells but does not extract the mitochondrial double membrane. Thus the kinetoplast, mitochondrion and the TAC remain intact. Aqueous silver precipitation is initiated around the ultra-small gold particles to enhance the label. Samples are then fixed and embedded using standard protocols [11]. Thin section micrographs of such experiments provided data that confirmed the remarkable Mab 22 recognition of the TAC exclusion zone. This data also confirmed that Mab 22 label is minimum on the basal bodies but almost exclusively located on 10 nm filaments subtending the basal bodies. These fibres are present in the cytoplasm and link the basal bodies to the outer mitochondrion membrane (Figure 1G–J). Figures 1G–J illustrates that our electron microscopy protocol allows visualisation and immunolabelling of basal body cytoskeleton structures without the extraction of mitochondrion membranes.

**Figure 2:**
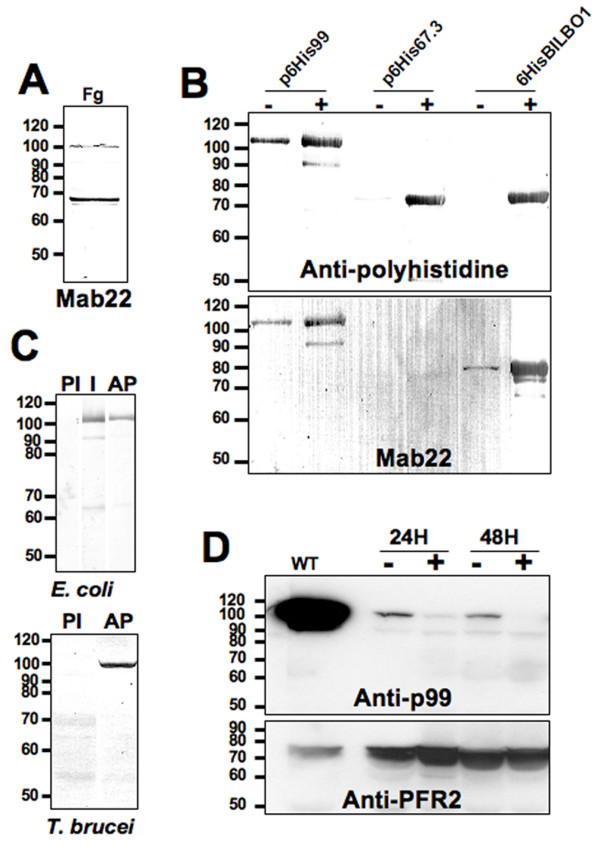
**Western blots**. A. Flagellar proteins (5.10^8 ^flagella, Fg) probed with Mab 22. B. N-terminal Histidine-tagged proteins expressed in *E. coli *(2.10^7 ^bacteria/well) and probed with anti-polyhistidine (upper panel) and Mab 22 (lower panel). Non-induced bacteria (-), 3 hr IPTG induction (+). C. Specificity of the affinity purified polyclonal anti-p99. In upper and lower panels PI: Pre-immune serum 1:1,000. I: Immune serum 1:500, AP: affinity purified immune serum 1:1000. WC: *T. brucei *whole cells (10^7^/well). D. p99 RNAi whole cells (4.10^7^/well) probed with the affinity purified immune serum anti-p99 1:500 and anti-PFR2 L8C4 monoclonal antibody (1:1,000) as loading control. Non-induced cells (-), Induced cells (+), Wild-type cells (WT).

Western blot data showed that MAb 22 blots to a 99 kDa protein and a 67–70 kDa protein (Figure 2A and 2B lower panel). After excising, from a SDS-PAGE gel, protein bands corresponding to the relative molecular masses recognized by Mab 22, we used mass spectrometry, to identify three proteins. These proteins were a 67.3 kDa, a 69 kDa and a 99 kDa protein (GeneDB corresponding accession numbers are Tb11.01.3960, Tb927.8.6660 and Tb11.01.2310 respectively). Their corresponding genes were cloned, tagged with a six histidine tag, expressed in *E. coli *and probed by Western blot with an anti-histidine antibody (Figure 2B upper panel). Mab 22 was positive by Western blotting on bacterial expressed recombinant 69 kDa and 99 kDa proteins but not (or extremely weak) for the 67.3 kDa protein (Figure 2B lower panel).

**Figure 3:**
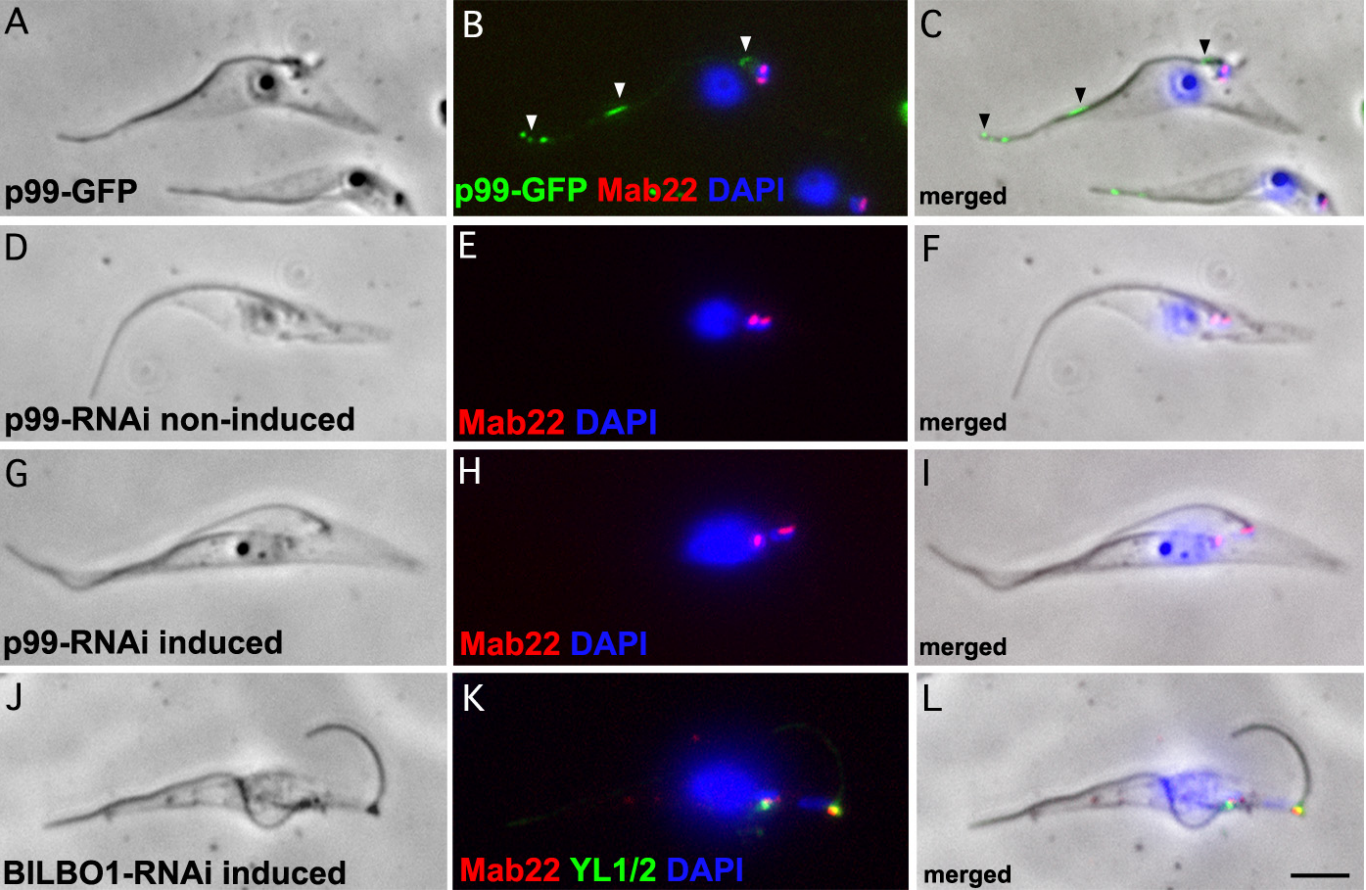
**GFP tagged and immunofluorescence on RNAi knockdown cells**. A-C. 48 h induced p99-GFP expressing cytoskeletons (green) probed with Mab 22 (red) and DAPI (Blue). We made a GFP tagged protein because the rabbit antibody raised to the 99 kDa was positive by Western blots on *E. coli *expressed purified proteins and trypanosome cells but was not detectible by immunofluorescence. D-F. Non-induced p99 RNAi cytoskeletons probed with Mab 22 and DAPI (Blue). G-I. 48 h induced p99 RNAi cytoskeletons probed by Mab 22 and DAPI (Blue). K-L. BILBO1 24 h induced RNAi cytoskeletons probed with Mab 22 and YL1/2 and DAPI (Blue). Note that the Mab 22 antibody signal remains present after probing p99 and BILBO1 RNAi induced knockdown cytoskeletons indicating that the gene expressing the TAC protein is neither p99 nor BILBO1. In A-L scale bar is 5 μm.

GFP tagging of the 99 kDa protein (now called p99) showed a weak, non-uniform axonemal signal rather than a basal body related signal (Figure 3A–C, see arrowheads in 3 B and C). We made a GFP tagged protein because the rabbit antibody raised to the 99 kDa was positive by Western blots on *E.coli *expressed purified proteins and trypanosome cells but was not detectible by immunofluorescence. In contrast, the 69 kDa band was identified as a flagellar pocket collar protein now named BILBO1. BILBO1 is a protein that is part of a structure required for the biogenesis of the flagellar pocket. RNAi knockdown of BILBO1 prevents new flagellar pocket biogenesis, blocks cell division, prevents new flagella attachment to the cell body, stimulates the formation of a large number of endo-exocytosis related vesicles and is lethal. The functional analysis, RNAi knockdown, GFP tagging and phenotype characterisation of BILBO1 is published elsewhere [10].

In cells induced for RNAi knockdown of the p99 and the BILBO1 protein, the Mab 22 signal remained present and was always observed at the basal bodies (Figure 3G–I and 3J–L). Knockdown of p99 was confirmed using the affinity purified polyclonal serum by Western blotting, which shows the loss of p99 after 48 hours of induction (see Figure 2C and 2D). The only phenotype observed after p99 knockdown was a minor reduction of cell culture growth (not shown). We also noticed that wild-type cells had a much higher concentration of p99 protein than non-induced p99 cells. We cannot explain this, but suggest that the reduction of protein in non-induced cells is most probably due to leakage of the inducible RNAi system (see Figure 2D). Our data suggested that the 99 kDa protein was most likely recognised by Mab 22 due to strong non-specific binding on Western blots and, that the major proteins recognised by Mab 22 using immunofluorescence are basal body-exclusion-zone TAC filaments and BILBO1. However, we cannot clearly explain the Mab 22 binding to both the BILBO1 and the 99 kDa proteins on WB since BILBO1 protein was recognised by immunofluorescence and on Western blots whereas the 99 kDa protein was identified only on Western blots. The recognition of the TAC protein appears to be restricted to conformational epitopes of native proteins and these epitopes are not conserved in SDS-PAGE.

During the replication of the kDNA the kinetoplast increases in size and undergoes initial segregation during the early stages of replication. In Figure 1J, an example of a kinetoplast in early S phase is observed. Because the mitochondrion membranes remain intact using our immuno-electron microscopy protocol, we note that interestingly Mab 22 positive fibres are observed at the flagellum face of the replicating kinetoplast between the mitochondrion membranes and both sets of basal bodies (old flagellum and new flagellum) during S phase. This data indicates the strong likelihood that the exclusion-zone filaments are present throughout the cell cycle.

## Discussion

These studies show that the Mab 22 positive exclusion-zone filaments are indeed present throughout the cell cycle including kinetoplast S phase. The kinetoplasts are segregated by basal body separation and thus implicate the Mab 22 positive fibres in kinetoplast segregation [4,16]. This data also shows that these exclusion zone filaments remain (directly or indirectly) in contact with replicating kDNA. Since this data was obtained from extracted cells it proves the existence of structural, detergent resistant, linkages between the kinetoplast and flagellum cytoskeleton. Recent research has shown the importance of correct function of the TAC [9] and thus a novel exclusion zone marker would be of importance in future studies of the TAC.

Although Mab 22 recognised 10 nm fibres of the TAC, our mass spectrometry analysis did not identify the genes encoding the fibres. Because these gene(s) are not known we can only make the postulation that the Mab 22 positive fibres are directly involved in kinetoplast segregation. However, Mab 22 does provide a novel marker for the presence of the exclusion-zone filaments.

Furthermore, the Mab 22 monoclonal will be useful in the investigation of the kDNA segregation process because recent studies show that RNAi knockdown of the only other known TAC protein, p166, initiates loss of kinetoplast segregation function [9]. It is not clear whether this loss of function may influence the biogenesis of other TAC components but Mab 22 would be a key reagent in testing this hypothesis. Mab 22 will therefore also be an excellent marker to test for coordination of TAC segregation *per se*. Mab 22 has been used previously as a control for basal body localisation [17]. However, this antibody was in the initial stages of production. It was considered as a simple basal body marker and was not characterised at the electron microscope level.

A number of key biological issues related to kDNA organization remain unresolved, particularly in terms of kDNA segregation. Since the replication network is also in the early stages of segregation the question arises as to how the flagella remains attached to the kDNA whilst the network is undergoing "S" phase or during kinetoplast remodelling? Recent studies have described a "hitherto unrecognized, intra-mitochondrial, filamentous structure that is rich in basic proteins and links the kDNA discs during their segregation" [18]. The authors show that two distinct sub-domains are present within the kineto-flagellar zone (a sub-compartment of the TAC) and that the unilateral filaments are composed of distinct inner and outer filaments. It is likely that replication coordination is linked to these structures and temporally linked to the development of the cytoskeleton.

## Conclusion

In this study we show that the antigen(s) recognised by Mab 22 are the first TAC exclusion-zone fibres to be identified. We demonstrate that these fibres are cytoskeletal and, insensitive to extraction by high concentrations of non-ionic detergent. We also illustrate that these Mab 22 positive exclusion-zone fibres extend from the proximal region of basal bodies and bind to the outer mitochondrial membrane. For future studies the more complex and challenging issues are to identify the genes encoding these fibres and carry out functional studies, determine how the kinetoplast is condensed and attached to the inner mitochondrial membrane and understand how the kinetoplast is temporally and spatially controlled.

## Abbreviations

Tripartite Attachment Complex: TAC.

## Competing interests

The authors declare that they have no competing interests.

## Authors' contributions

MB did the western blotting, the gene identification, gene cloning and some of the immunofluorescence. NL did some of the immunofluorescence and western blotting. MD did the EM work and developed the protocol for the EM. DR wrote the manuscript, developed the protocol for the EM work and did some of the immunofluorescence work.
